# Supplementary material for: The interactome of intact mitochondria by cross-linking mass spectrometry provides evidence for coexisting respiratory supercomplexes
Source: Mol Cell Proteomics. 2017 Dec 8;17(2):216–32. doi: 10.1074/mcp.RA117.000470 (PMC5795388; doi:10.1074/mcp.RA117.000470)
Supplement: Supplemental Data [file supp_RA117.000470_133922_0_supp_23978_fzffwf.zip › spectra_annotation/mito_DR_spectra_annotation/84-1-8-1-27-1.pdf]

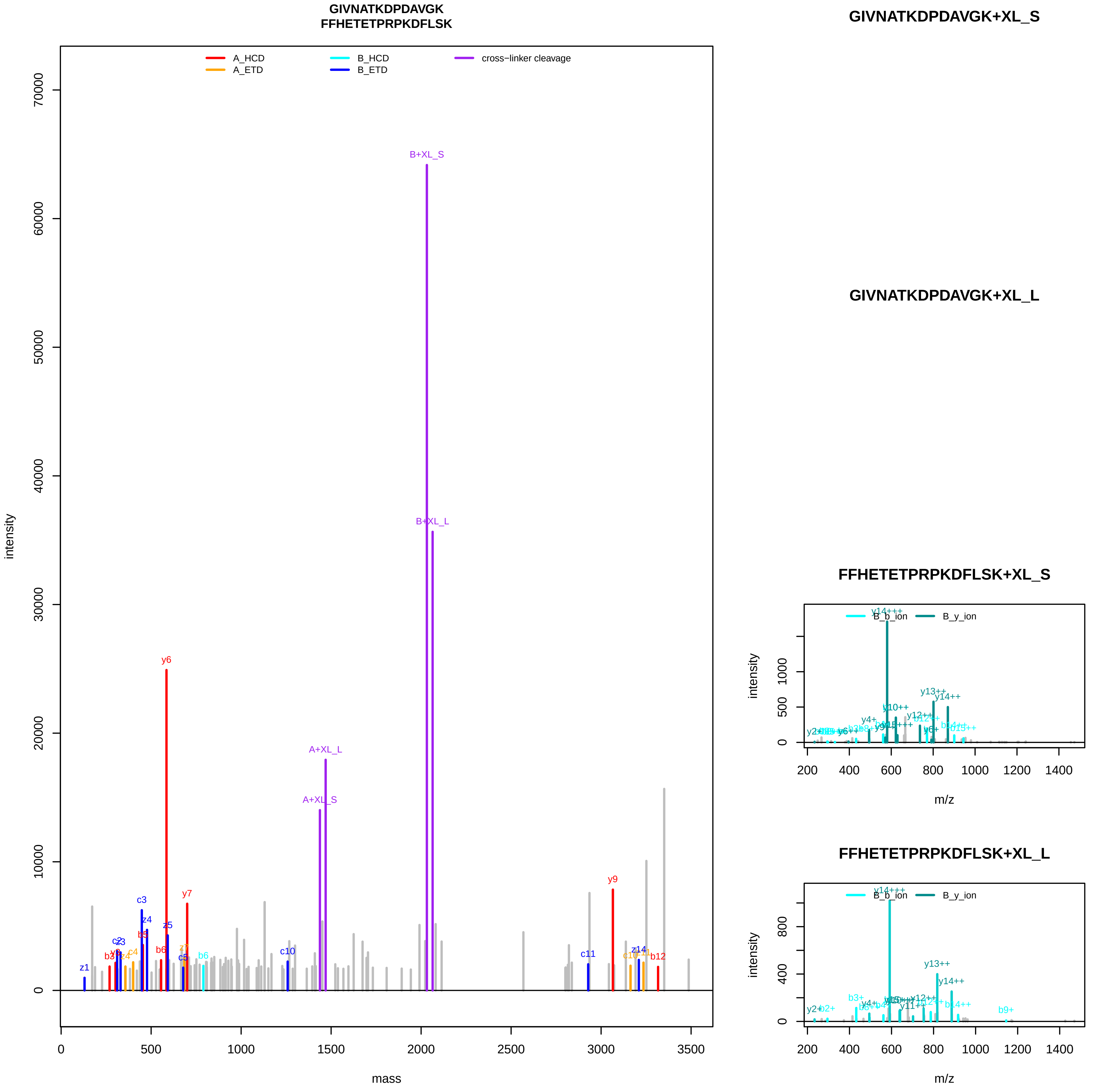
GIVNATKDPDAVGK
FFHETETPRPKDFLSK
A_HCD
A_ETD
B_HCD
B_ETD
cross−linker cleavage
B+XL_S
B+XL_L
A+XL_L
A+XL_S
y6
y7
y9
b12
c3
z4
z5
c5
b6
c10
c11
z14
z1
intensity
mass
GIVNATKDPDAVGK+XL_S
GIVNATKDPDAVGK+XL_L
FFHETETPRPKDFLSK+XL_S
FFHETETPRPKDFLSK+XL_L
B_b_ion
B_y_ion
y13++
y14++
y10++
y14+++
b9+
b3+
y2+
b2+
m/z
